# Disparities in length of life across developed countries: measuring and decomposing changes over time within and between country groups

**DOI:** 10.1186/s12963-016-0094-0

**Published:** 2016-08-11

**Authors:** Sergey Timonin, Vladimir M. Shkolnikov, Domantas Jasilionis, Pavel Grigoriev, Dmitry A. Jdanov, David A. Leon

**Affiliations:** 1 Institute of Demography, National Research University Higher School of Economics, Myasnitskaya Street 20, 101000 Moscow, Russia; 2 Center for Demographic Research, New Economic School, Nakhimovskii Prospekt 47, 117418 Moscow, Russia; 3 Laboratory of Demographic Data, Max Planck Institute for Demographic Research, Konrad-Zuse-Strasse 1, 18057 Rostock, Germany; 4 Department of Non-communicable Disease Epidemiology, London School of Hygiene and Tropical Medicine, Keppel Street, London, WC1E 7HT UK; 5 Department of Community Medicine, The Arctic University of Norway, Postboks 6050 Langnes, 9037 Tromsø, Norway

**Keywords:** Mortality disparities, Developed countries, East–West gap in life expectancy, Decomposition, Stepwise replacement algorithm

## Abstract

**Background:**

Over the past half century the global tendency for improvements in longevity has been uneven across countries. This has resulted in widening of inter-country disparities in life expectancy. Moreover, the pattern of divergence appears to be driven in part by processes at the level of country groupings defined in geopolitical terms. A systematic quantitative analysis of this phenomenon has not been possible using demographic decomposition approaches as these have not been suitably adapted for this purpose. In this paper we present an elaboration of conventional decomposition techniques to provide a toolkit for analysis of the inter-country variance, and illustrate its use by analyzing trends in life expectancy in developed countries over a 40-year period.

**Methods:**

We analyze trends in the population-weighted variance of life expectancy at birth across 36 developed countries and three country groups over the period 1970–2010. We have modified existing decomposition approaches using the stepwise replacement algorithm to compute age components of changes in the total variance as well as variance between and within groups of Established Market Economies (EME), Central and Eastern Europe (CEE), and the Former Soviet Union (FSU). The method is generally applicable to the decomposition of temporal changes in any aggregate index based on a set of populations.

**Results:**

The divergence in life expectancy between developed countries has generally increased over the study period. This tendency dominated from the beginning of 1970s to the early 2000s, and reversed only after 2005. From 1970 to 2010, the total standard deviation of life expectancy increased from 2.0 to 5.6 years among men and from 1.0 to 3.6 years among women. This was determined by the between-group effects due to polarization between the EME and the FSU. The latter contrast was largely fueled by the long-term health crisis in Russia. With respect to age, the increase in the overall divergence was attributable to between-country differences in mortality changes at ages 15–64 years compared to those aged 65 and older. The within-group variance increased, especially among women. This change was mostly produced by growing mortality differences at ages 65 and older.

**Conclusions:**

From the early 1970s to the mid-2000s, the strong divergence in life expectancy across developed countries was largely determined by the between-group variance and mortality polarization linked to the East–West geopolitical division.

## Introduction

The lengthening of the human lifespan is one of the most remarkable achievements of human civilization [1–3]. Over the past 60 years, global life expectancy has greatly increased. However, life expectancy gains have been uneven across countries, and during the last decades of the 20th century, inter-country disparities expanded [4, 5]. While life expectancy increased steadily in most countries, some countries and regions had larger gains than others, and some even experienced mortality reversals [6–10]. Among developed countries, the most significant mortality reversals occurred in Eastern Europe. Starting in the late 1960s, this region experienced several decades of rising mortality in particular from cardiovascular diseases, alcohol-related and external causes of death (i.e., injuries, poisonings, and violence). However, at the very end of the 1980s, around the time of the fall of the Berlin Wall, a more favorable dynamic of decreasing mortality emerged in Central and Eastern Europe. Some years later, in the mid-1990s, a similar decline in mortality took place in the former Soviet Baltic states. Finally, in the mid-2000s, favorable changes started in the Slavic countries of the former Soviet Union [11–17].

Although in the established market economies (EME) life expectancy has been rising continuously, there has also been variation in the size of these increases, as certain countries (Japan, Spain, Italy, France) have improved their relative positions, while others (Denmark, the United States, the Netherlands) have lost their former advantages.

A number of studies have carried out classic decomposition analyses of life expectancy changes in single countries of Europe and North America with the goal of identifying the age- and cause-specific changes in mortality that are most responsible for the changes in longevity. These have found that in Russia, Ukraine, Belarus, the Baltic countries, Hungary, Poland, and other countries of Eastern Europe, the declining or stagnating life expectancy levels over the 1970s–1980s were largely attributable to rising mortality among young and middle-aged adults, combined with a lack of mortality improvements among the elderly [6, 13, 18–21]. It has also been shown also that the speed at which longevity was increasing was higher in some of the advanced countries and lower in others due to variation in mortality improvements among the elderly [22–24].

Over the past 15 years studies looking at mortality and life expectancy changes in single countries or comparing trends among a few countries were complemented by analyses of mortality trends across all or most of the countries of the world [4, 8, 25, 26]. The latter studies by Moser et al. [4] and by Smits and Monden [8] used single summary measures of inequality to quantify changes in the amount of worldwide length of life disparity. These studies provided an instrument for an objective detection of transitions from mortality convergence to mortality divergence. This sort of transition has been conceptualized by Meslé and Vallin’s convergence-divergence theory and is attributed to the emergence of new epidemiological challenges which are addressed at first by a few vanguard countries and only later (after some years or even decades) by other countries [27, 28].

The conventional decomposition analyses discussed above have been used for investigating components and driving forces of changes in life expectancy in single countries. In the present study, we show how these methods can be adapted and elaborated in order to analyze changes in summary measures of inter-country disparity based on data from many countries, and then illustrate their use by looking at trends in mortality in 36 developed countries over the period 1970–2010.

The technical details of this method are provided in the next section, but for the sake of clarity we provide in the remainder of the Introduction a high-level summary of our approach. We use the population-weighted cross-country variance as a measure of the amount of inter-country disparity. Because this measure takes into account the population size of each country, it has the advantage of reflecting the overall *public health burden* produced by the disparity. The weighted disparity measure expresses not only the lifetime differences among countries and country groups, but also how many people in different countries experience longer or shorter lifetimes. However, to see how the weighting influences the results, we provide results of alternative calculations without population weighting.

Our empirical analysis includes decomposition of changes in the life expectancy dispersion across a set of 36 developed countries using the general stepwise replacement algorithm [29]. We further substantiate the converge-divergence theory by new information about components of the life expectancy divergence of the 1970s–1990s and convergence of the 2000s. In particular, for the first time we evaluate the importance of changes in the amount of length of life disparity: 1) of the former East–West geopolitical divide, and specifically of the country groups of established market economies (EME), Central and Eastern Europe (CEE), and the former Soviet Union (FSU); 2) of the three principal age groups (childhood, midlife, and old ages); 3) of mortality and population composition.

## Data and methods

### Data

We use the Human Mortality Database (HMD) data [30] on deaths and population exposure by sex and age (0, 1–4, 5–9, 10–14,…, 95+) for 36 developed countries and regions (see Appendix 1 for the list of countries) over the period 1970 to 2010.^1^ The starting point approximates the beginning of a new phase of longevity divergence [28], while the end point is the most recent year for which data for the majority of the countries are available (see Appendix 2 for the country-specific life expectancy values).

Between 1970 and 2010, the total population of the countries under study increased from 1.0 to 1.2 billion. Over this period, the share of the total population in the EME countries rose from 71 to 75 %, the share of the total population in the CEE countries decreased from 9 to 8 %, and the share of the total population in the FSU countries declined from 20 to 17 % (see Appendix 3 for the country-specific population sizes).

The use of the HMD ensures that the mortality data are of high quality up to advanced ages, which is important due to the substantial contributions of old-age mortality to the changes in longevity during the period under study.

## Methods

### Measuring inter-country disparity

Consider a set of populations *i* (*i* = 1, 2,…, *n*) split by age *x*. In year *t*, this set can be described by the matrix of death rates **M**(*t*) = [*m*_*x*,*i*_(*t*)] and by the matrix of population exposures **P**(*t*) = [*p*_*x*,*i*_(*t*)]. In matrix **M**(*t*), each column *M*_*i*_ (*t*) is a vector of age-specific death rates in country *i* at time *t*. The corresponding life expectancy at birth *e*_0_, as a function of vector *M*_*i*_ (*t*), is denoted as *e*_0_(*M*_*i*_(*t*)). This function produces *e*_0_ values through computing life tables from death rates *m*_*x*,*i*_(*t*).

The aggregate scalar index for expressing central tendency in a group of *n* populations at time *t* is the population-weighted average length of life:

1 $$ \overline{e_0(t)}={\displaystyle {\sum}_i{\pi}_i(t){e}_0\left({M}_i(t)\right),} $$

with the population weights being ${\pi}_i(t)=\frac{{\displaystyle {\sum}_x}{p}_{x,i}(t)}{{\displaystyle {\sum}_i}{\displaystyle {\sum}_x}{p}_{x,i}(t)}.$

Following Edwards [7], the *population-weighted cross-country variance* is used as an aggregate index of the lifetime disparity across countries:

2 $$ Var(t)={\displaystyle {\sum}_i{\pi}_i(t){\left[{e}_0\left({M}_i(t)\right)-\overline{e_0(t)}\right]}^2}. $$

This dispersion measure closely correlates with other measures of inequality, and has the advantage of being analytically decomposable into within- and between-group partitions. The split of the total variance into variance within and between country groups will be used in our study. While the total variance measures the amount of dispersion due to all potential factors, the between-group variance measures the contribution of the factors used to define the groups. The within-group variance measures dispersion caused by factors acting within the groups.

The units of variance are years squared, unlike life expectancy which is in units of years. For this reason, we also calculate *the standard deviation* as a measure of disparity measured in units of years:

3 $$ StD\ (t)={\left(Var(t)\right)}^{1/2}. $$

### Decomposition problem and its solution

Consider an aggregated measure *F* (equal to $\overline{e_0}$ or *Var* or *StD* or another index computed from the length of life distribution across countries) defined (according to equations (1) to (3)) as a function of matrices **M** and **P**, with its values changing between times *t*_0_ and *T*. The decomposition task is to compute additive components of the total change *F*(*T*)-*F*(*t*_0_) produced by age-specific changes in countries’ mortality rates (M-effects) and in countries’ population weights (P-effects).

The conventional decomposition equations which were (independently from each other) deduced in the 1980s by Andreev [31], Arriaga [32], and Pressat [33] cannot be used for completing this decomposition task, since these equations decompose a change only in the life expectancy and only in a single population between two different time points or between a pair of populations at one time point. However, the general stepwise replacement algorithm can be employed for completing the task. This method can be used for decompositions involving various output indexes calculated from data on more than one population (see Appendix 4 for a summary of the algorithm).

In our earlier work, we used the stepwise replacement algorithm for decomposition of changes in life expectancy of the total population between two time points into age-specific contributions of: 1) mortality within educational groups and 2) educational structure of the population [29]. The same method was subsequently applied to decompose changes in the total population’s length of life by occupational and marital status groups [34–36]. In all these earlier decompositions, the replacement was running in the ascending order across ages, but within each age all possible replacement sequences (related to educational, occupational, or marital status groups) were realized followed by averaging components corresponding to these sequences.

In the present study, the stepwise replacement algorithm is used for decomposition of temporal change in measure *F* by: 1) age, mortality, and population composition; 2) by age and country group. Applying the method to the set of populations, the age components and their mortality and population-composition parts (M- and P-effects) can be obtained by running a sequence of replacements of the elements *m*_*x*,*i*_(*t*_0_) and *p*_*x*,*i*_(*t*_0_) by the elements *m*_*x*,*i*_(*T*) and *p*_*x*,*i*_(*T*). In matrices **M** and **P**, the replacement progresses from the youngest age zero to the oldest age 95+. Let **M**^[*x*]^(*t*_0_, *T*) and **P**^[*x*]^(*t*_0_, *T*) be matrices containing elements *m*_*y*,*i*_(*T*) and *p*_*y*,*i*_(*T*) at ages 0 ≤ *y* < *x* and elements *m*_*y*,*i*_(*t*_0_) and *p*_*y*,*i*_(*t*_0_) at ages ≥ *x*:

4 $$ {\mathbf{M}}^{\left[\boldsymbol{x}\right]}\left({t}_0,T\right)=\left[\begin{array}{cccccc}\hfill {}_1{m}_{0,1}(T)\hfill & \hfill {}_1{m}_{0,2}(T)\hfill & \hfill \cdots \hfill & \hfill {}_1{m}_{0,i}(T)\hfill & \hfill \cdots \hfill & \hfill {}_1{m}_{0,n}(T)\hfill \\ {}\hfill \underset{\cdots }{{}_4{m}_{1,1}(T)}\hfill & \hfill \underset{\cdots }{{}_4{m}_{1,2}(T)}\hfill & \hfill \cdots \hfill & \hfill \underset{\cdots }{{}_4{m}_{1,i}(T)}\hfill & \hfill \cdots \hfill & \hfill \underset{\cdots }{{}_4{m}_{1,n}(T)}\hfill \\ {}\hfill {}_5{m}_{x-5,1}(T)\hfill & \hfill {}_5{m}_{x-5,2}(T)\hfill & \hfill \begin{array}{l}\cdots \\ {}\cdots \end{array}\hfill & \hfill {}_5{m}_{x-5,i}(T)\hfill & \hfill \begin{array}{l}\cdots \\ {}\cdots \end{array}\hfill & \hfill {}_5{m}_{x-5,n}(T)\hfill \\ {}\hfill \underset{\cdots }{{}_5{m}_{x,1}\left({t}_0\right)}\hfill & \hfill \underset{\cdots }{{}_5{m}_{x,2}\left({t}_0\right)}\hfill & \hfill \begin{array}{l}\cdots \\ {}\cdots \end{array}\hfill & \hfill \underset{\cdots }{{}_5{m}_{x,i}\left({t}_0\right)}\hfill & \hfill \begin{array}{l}\cdots \\ {}\cdots \end{array}\hfill & \hfill \underset{\cdots }{{}_5{m}_{x,n}\left({t}_0\right)}\hfill \\ {}\hfill {}_{\infty }{m}_{95,1}\left({t}_0\right)\hfill & \hfill {}_{\infty }{m}_{95,2}\left({t}_0\right)\hfill & \hfill \cdots \hfill & \hfill {}_{\infty }{m}_{95,i}\left({t}_0\right)\hfill & \hfill \cdots \hfill & \hfill {}_{\infty }{m}_{95,n}\left({t}_0\right)\hfill \end{array}\right], $$

$$ {\mathbf{M}}^{\left[0\right]}\left({t}_0,T\right)=\mathbf{M}\left({t}_0\right), $$

5 $$ {\mathbf{P}}^{\left[\boldsymbol{x}\right]}\left({t}_0,T\right)=\left[\begin{array}{cccccc}\hfill {}_1{p}_{0,1}(T)\hfill & \hfill {}_1{p}_{0,2}(T)\hfill & \hfill \cdots \hfill & \hfill {}_1{p}_{0,i}(T)\hfill & \hfill \cdots \hfill & \hfill {}_1{p}_{0,n}(T)\hfill \\ {}\hfill \underset{\cdots }{{}_4{p}_{1,1}(T)}\hfill & \hfill \underset{\cdots }{{}_4{p}_{1,2}(T)}\hfill & \hfill \cdots \hfill & \hfill \underset{\cdots }{{}_4{p}_{1,i}(T)}\hfill & \hfill \cdots \hfill & \hfill \underset{\cdots }{{}_4{p}_{1,n}(T)}\hfill \\ {}\hfill {}_5{p}_{x-5,1}(T)\hfill & \hfill {}_5{p}_{x-5,2}(T)\hfill & \hfill \begin{array}{l}\cdots \\ {}\cdots \end{array}\hfill & \hfill {}_5{p}_{x-5,i}(T)\hfill & \hfill \begin{array}{l}\cdots \\ {}\cdots \end{array}\hfill & \hfill {}_5{p}_{x-5,n}(T)\hfill \\ {}\hfill \underset{\cdots }{{}_5{p}_{x,1}\left({t}_0\right)}\hfill & \hfill \underset{\cdots }{{}_5{p}_{x,2}\left({t}_0\right)}\hfill & \hfill \begin{array}{l}\cdots \\ {}\cdots \end{array}\hfill & \hfill \underset{\cdots }{{}_5{p}_{x,i}\left({t}_0\right)}\hfill & \hfill \begin{array}{l}\cdots \\ {}\cdots \end{array}\hfill & \hfill \underset{\cdots }{{}_5{p}_{x,n}\left({t}_0\right)}\hfill \\ {}\hfill {}_{\infty }{p}_{95,1}\left({t}_0\right)\hfill & \hfill {}_{\infty }{p}_{95,2}\left({t}_0\right)\hfill & \hfill \cdots \hfill & \hfill {}_{\infty }{p}_{95,i}\left({t}_0\right)\hfill & \hfill \cdots \hfill & \hfill {}_{\infty }{p}_{95,n}\left({t}_0\right)\hfill \end{array}\right]. $$

$$ {\mathbf{P}}^{\left[0\right]}\left({t}_0,T\right)=\mathbf{P}\left({t}_0\right)\ . $$

One step in the replacement sequence includes replacement pertaining to one elementary age group [*x*, *x* + *a*). The rows corresponding to this age group with elements *m*_*x,i*_(*t*_0_) and *p*_*x,i*_(*t*_0_) in the two matrices **M** and **P** should be replaced by respective elements *m*_*x,i*_(*T*) and *p*_*x,i*_(*T*) and the effect of these replacement on the value of *F* should be computed. Using the notation given in equations (4) and (5), the respective M- and P-effects are:

6 $$ {\varDelta}_M^x\left({t}_0,T\right)=\frac{1}{2}\left\{\left[F\left({\mathbf{M}}^{\left[x+a\right]},{\mathbf{P}}^{\left[x\right]}\right)-F\left({\mathbf{M}}^{\left[x\right]},{\mathbf{P}}^{\left[x\right]}\right)\right]+\left[F\left({\mathbf{M}}^{\left[x+a\right]},{\mathbf{P}}^{\left[x+a\right]}\right)-F\left({\mathbf{M}}^{\left[x\right]},{\mathbf{P}}^{\left[x+a\right]}\right)\right]\right\}, $$

7 $$ {\varDelta}_P^x\left({t}_0,T\right)=\frac{1}{2}\left\{\left[F\left({\mathbf{M}}^{\left[x\right]},{\mathbf{P}}^{\left[x+a\right]}\right)-F\left({\mathbf{M}}^{\left[x\right]},{\mathbf{P}}^{\left[x\right]}\right)\right]+\left[F\left({\mathbf{M}}^{\left[x+a\right]},{\mathbf{P}}^{\left[x+a\right]}\right)-F\left({\mathbf{M}}^{\left[x+a\right]},{\mathbf{P}}^{\left[x\right]}\right)\right]\right\}. $$

The component corresponding to change in the elementary age group [*x*, *x* + *a*] is a sum of the M- and P-effects:

8 $$ {\varDelta}^x\left({t}_0,T\right)={\varDelta}_M^x\left({t}_0,T\right)+{\varDelta}_P^x\left({t}_0,T\right). $$

Finally, the total change in the function *F* between times *t*_0_ and *T* is:

9 $$ F(T)-F\left({t}_0\right)={\displaystyle {\sum}_x{\varDelta}^x}\left({t}_0,T\right). $$

The approach to the decomposition by age and country (or country group) is similar to the one used for decomposition by age, mortality, and population composition (equations (4)-(9)). Again, one has to carry out a sequence of replacements of rows (ages) in matrices **M** and **P**. However, instead of replacing entire rows in the two matrices, it would be necessary to replace parts of these rows corresponding to certain countries or country groups.

Let us consider a super-simple case with two populations and one age group only. Accordingly, the function *F*(*t*) depends on four elements: *m*_1_(*t*), *m*_2_(*t*) and *p*_1_(*t*), *p*_2_(*t*). Each elementary age component ∆ has to be presented as a sum of two country components:

10 $$ F(T)-F\left({t}_0\right)={\varDelta}_1+{\varDelta}_2. $$

The component produced by population 1 is to be computed as an effect of replacements *m*_1_(*T*) → *m*_1_(*t*) , *p*_1_(*T*) → *p*_1_(*t*). Here, we should take into account two possible replacement sequences:

11 $$ {\varDelta}_{1(1)}=F\left({m}_1(T),\ {m}_2(t),\ {p}_1(T),\ {p}_2(t)\right)-F\left({m}_1(t),\ {m}_2(t),\ {p}_1(t),\ {p}_2(t)\right); $$

12 $$ {\varDelta}_{1(2)}=F\left({m}_1(T),\ {m}_2(T),\ {p}_1(T),\ {p}_2(T)\right)-F\left({m}_1(t),\ {m}_2(T),\ {p}_1(t),\ {p}_2(T)\right). $$

The final component produced by country 1 should is the average of components produced by the two sequences:

13 $$ {\varDelta}_1=\frac{1}{2}\left({\varDelta}_{1(1)}+{\varDelta}_{1(2)}\right) $$

Accordingly, the component produced by population 2 is calculated as an effect of replacements *m*_2_(*T*) →*m*_2_(*t*) , *p*_2_(*T*) → *p*_2_(*t*) on function *F*. These effects are calculated similarly to equations (10)-(13).

For the three country-groups within each age, the group-specific components have to be completed for all replacement sequences and the results have to be average over these sequences. A more systematic description of the method for the decomposition by age and country or country group is given in Appendix 5.

## Results

### Life expectancy trends and cross-country disparity

**Fig. 1:**
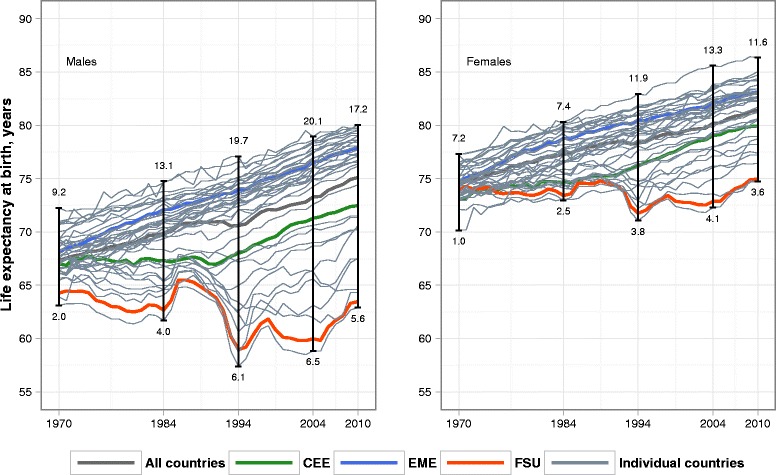
Life expectancy at birth for countries and country groups by sex in 1970–2010. Note: max-min range in life expectancy is shown above the trends; population-weighted standard deviation in life expectancy is shown below the trends

Figure 1 shows the time trends in life expectancy at birth for the 36 developed countries included, the three country groups, and the aggregate of all 36 countries combined. Table 1 summarizes these trends at a level of country groups. During the period 1970–2010, the overall population-weighted average life expectancy increased at an annual average rate of 0.21 and 0.17 years for males and females, respectively. However, there was substantial variation across the three groups. Robust and sustained improvements were seen in the EMEs (0.24 and 0.20 years annually for males and females, respectively), while less favorable changes were observed in the CEE (0.15 and 0.18 years) and the FSU (−0.02 and 0.03 years) groups. In fact, the group-level averages for the CEE and (some of) the FSU countries obscures the fact that for much of the period, life expectancy was deteriorating in the FSU group, and was stagnating in the CEE group (see Appendix 2 and Appendix 3 for country-specific life expectancies and population weights in 1970, 1984, 1994, 2004, and 2010, respectively). Between 1970 and 2010, the range (maximum minus minimum) across the entire set of countries increased by 8.0 years for males and 4.4 years for females, and the *StD* nearly tripled for both sexes.

**Table 1:** Life expectancy at birth and measures of variance for the entire set of countries and for country groups in selected years (in years)

	Males					Females				
	1970	1984	1994	2004	2010^a^	1970	1984	1994	2004	2010^a^
*Overall life expectancy, including:*	*67.31*	*69.85*	*70.59*	*73.27*	*75.10*	*74.42*	*77.29*	*78.33*	*80.18*	*81.41*
EME	68.18	72.08	73.91	76.56	77.90	74.75	78.75	80.43	82.18	83.13
CEE	67.00	67.32	67.98	71.27	72.46	73.06	74.68	76.19	79.03	79.94
FSU	64.26	62.67	58.97	59.98	63.46	73.88	73.41	71.75	72.86	74.95
*Max-Min range between country groups*	*3.91*	*9.41*	*14.94*	*16.58*	*14.44*	*1.69*	*5.34*	*8.68*	*9.32*	*8.18*
*Overall standard deviation, including:*	*2.04*	*4.00*	*6.09*	*6.45*	*5.55*	*1.00*	*2.47*	*3.77*	*4.06*	*3.57*
EME	1.28	1.37	1.48	1.39	1.31	0.91	1.00	1.46	1.99	1.84
CEE	1.01	1.37	1.67	2.11	2.24	0.39	0.66	1.14	1.57	1.51
FSU	1.71	1.44	2.36	1.88	1.09	0.67	0.74	1.07	1.11	0.79

Note: ^a^Years in the range 2008–2010 depending on availability (see Appendix 3)

As the direction and the magnitude of the variance changes varied with time, the observation period was divided into sub-periods according to the character of the changes. While the *StD* increased continuously and gradually between 1970 and 1984, it fluctuated between 1985 and 2004, decreasing briefly around 1986 and then rising to the highest levels observed in 1994. Between 1995 and 2004, the *StD* continued to fluctuate, albeit to a lesser degree, and ended the period slightly higher. Finally, during the last period of 2005–2010, the *StD* decreased somewhat.

These changes in the *StD* were largely attributable to the high degree of variability in life expectancy in the FSU, and especially in Russia, the largest of the FSU countries. In Russia, the first period of 1970–1984 was characterized by a gradual deterioration in health [37]. The second period of 1985–1994 began with a sudden decrease in mortality associated with Gorbachev’s anti-alcohol campaign followed by a sharp rise in the early 1990s, which coincided with the resumption of the widespread availability of alcohol and the implementation of painful political and economic reforms [38]. The third period of 1995–2004 included another episode of recovery followed by further deterioration in life expectancy after the economic crisis of 1998. In 2005–2010, life expectancy in Russia and the other FSU countries increased substantially, a development which has been attributed to economic growth, the reduction of harm from alcohol, and improvements in the health care system [39].

In the CEE countries, life expectancy stagnation during the communist era in the 1970s and 1980s was followed by significant improvement between 1994 and 2004. In 2005–2010, the progress in life expectancy in the CEE continued but at a somewhat slower pace.

Life expectancy improved far more in the EME group than in the FSU and the CEE groups. Between 1970 and 2010, the gap between the EME and FSU countries increased from 3.9 to 14.4 years for males and from 0.9 years to 8.2 years for females. However, a more detailed look at the life expectancy dynamics across time and individual countries within the EME group reveals some variation in the magnitude of lifetime gains, with slower progress in the United States, the largest country in the group.

Table 1 suggests that the country groups experienced quite different patterns of dispersion change. Within the EME group, there was an important difference between males and females. While for males *StD* did not change much, for females it doubled and in the 2000s substantially exceeded the corresponding male values. Within the CEE group, *StD* increased steeply and continuously, with values for males always exceeding those for females. From 1970 to the 2000s it doubled for males and nearly quadrupled for females. Looking at country data in Appendix 2 and Appendix 3, it is apparent that there was a growing contrast between the group leader (East Germany), the group mainstream (Czech Republic, Poland, and Slovakia), and the countries who lagged behind (Bulgaria and Hungary). Finally, males of the FSU group experienced the greatest *StD* variation across time with a steep rise from 1970 to the mid-1990s and a sharp drop in the 2000s. Among the FSU females, *StD* followed a broadly similar trajectory. Although FSU showed the largest gap between male and female *StD*s, females were broadly similar to males with respect to temporal changes. It is surprising that in spite of the StD elevation in the 1970s–1990s, in 2010 the male *StD* was lower and female *StD* did not differ much from the starting levels of 1970. This is attributable to the fact that Ukraine and Belarus have been losing their starting life expectancy advantage relative to Russia.

Importantly, *StD* values within the three country groups were very much lower than the overall *StD* across all countries. This suggests a great role of the between-group lifetime variance.

### Between- and within-group variance

**Fig. 2:**
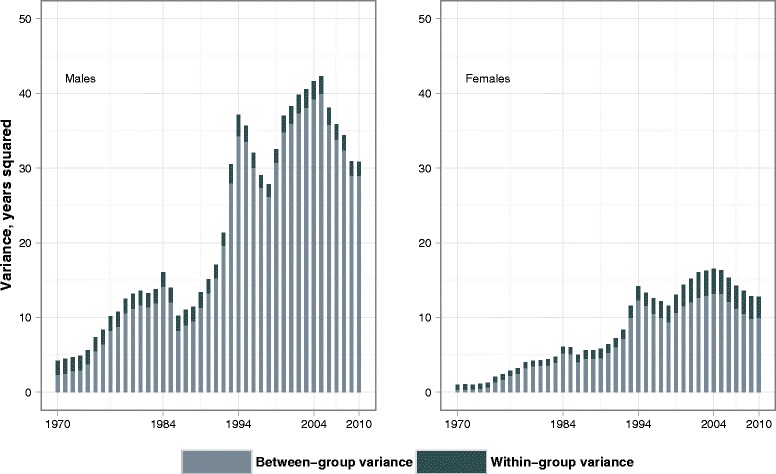
Between- and within-group components of life expectancy variance by sex in 1970–2010

Figure 2 further highlights the sharp increase in inter-country disparities from 1970 to 2004, which was interrupted twice by short episodes of increasing length of life in the FSU countries in the mid-1980s and the mid-1990s. It is also clear that this increase was almost entirely determined by the between-group variance. Whereas in the early 1970s there were moderate differences in the mortality levels of the three groups, in later decades these differences became much more pronounced, especially among men. Over the same period, however, the within-group variance was relatively stable. This suggests that *mortality polarization* linked to the former East–West geopolitical divide played an important role. The degree of polarization weakened slightly during the last five years of observation, but in 2010 it was still very much higher than in 1970.

Table 2 provides further insights into the patterns of the between- and within-group variance. Among males the FSU group accounted for most (70–80 %) of the between-group variance over the whole observation period. Among females the FSU contribution to the between-group variance rose from around 20 % up to 70 % or more by the 1990s. Due to female life expectancy in the CEE countries being particularly low at the start of the period, the CEE contribution to the between-variance exceeded 50 % in the early 1970s but declined to about 2 % in 2010.

**Table 2:** Between- and within-group variance and its distribution by country groups in selected years

	Males					Females				
	1970	1984	1994	2004	2010^a^	1970	1984	1994	2004	2010^a^
*Total cross-country variance, years squared*	*4.18*	*16.03*	*37.12*	*41.61*	*30.83*	*0.99*	*6.09*	*14.18*	*16.51*	*12.77*
*Between-group variance, years squared*	*2.35*	*14.12*	*34.24*	*39.22*	*28.97*	*0.3*	*5.23*	*12.32*	*13.19*	*9.95*
EME, %	22.7	25.4	23.4	20.7	20.5	26.0	28.9	25.8	22.4	22.2
CEE, %	0.4	3.9	1.6	0.8	1.8	53.5	11.0	3.0	0.8	1.6
FSU, %	76.9	70.7	75.0	78.5	77.7	20.5	60.1	71.2	76.8	76.2
*Between-group standard deviation, years*	*1.53*	*3.76*	*5.85*	*6.26*	*5.38*	*0.55*	*2.29*	*3.51*	*3.63*	*3.16*
*Within-group variance, years squared*	*1.83*	*1.91*	*2.88*	*2.40*	*1.86*	*0.69*	*0.86*	*1.87*	*3.31*	*2.82*
EME, %	64.0	70.5	55.3	60.4	69.7	84.3	82.3	82.0	87.4	90.0
CEE, %	4.9	8.4	7.8	14.0	19.7	1.9	4.3	5.6	5.6	5.9
FSU, %	31.1	21.1	36.9	25.6	10.6	13.8	13.4	12.4	7.0	4.0
*Within-group standard deviation, years*	*1.34*	*1.38*	*1.66*	*1.53*	*1.34*	*0.81*	*0.92*	*1.36*	*1.79*	*1.63*

Note: ^a^Years in the range 2008–2010 depending on availability (see Appendix 3)

When we look at the total *within-group disparity* in life expectancy (second half of Table 2), a few interesting features stand out. First, the values of the within-group variance did not differ much between males and females. In fact, the female within-group variance exceeded the corresponding male values from the mid-1990s onward. Second, the EME group accounted for a large part of the total within-group variance, especially among females.

### Components of the variance change

This section presents the results of decompositions of the change in the population-weighted *StD*. As explained in the Methods, a change in an aggregate measure in a set of countries depends on population (P-effects) as well as mortality (M-effects). While changes in countries’ population weights were usually minor, mortality changes were more substantial and also varied considerably across countries. Thus one can expect that the M-effects would be much greater than P-effects, as confirmed in Table 3. Only after the mid-1990s is it possible to see some P-effects of the overall and of the between-group *StD* changes among males. These effects were predominantly negative as a result of the redistribution of the population in favor of countries with lower mortality (the EME countries). In the within-group *StD,* P-effects of changes were negligible.

**Table 3:** Contributions of population composition (P-effects) and mortality (M-effects) to changes in standard deviation by time periods (in years)

	Males				Females			
	1970–1984	1984–1994	1994–2004	2004–2010^a^	1970–1984	1984–1994	1994–2004	2004–2010^a^
*Total change in overall StD:*	*1.96*	*2.09*	*0.36*	*−0.90*	*1.47*	*1.30*	*0.30*	*−0.49*
P-effect	0.01	−0.04	−0.18	−0.10	0.00	−0.03	−0.08	−0.05
M-effect	1.95	2.12	0.54	−0.80	1.47	1.33	0.38	−0.44
*Total change in between-group StD:*	*2.23*	*2.09*	*0.41*	*−0.88*	*1.74*	*1.22*	*0.12*	*−0.48*
P-effect	0.01	−0.03	−0.18	−0.10	0.00	−0.03	−0.09	−0.05
M-effect	2.22	2.13	0.59	−0.78	1.74	1.26	0.21	−0.42
*Total change in within-group StD:*	*0.03*	*0.31*	*−0.15*	*−0.19*	*0.09*	*0.44*	*0.45*	*−0.14*
P-effect	0.00	−0.01	−0.02	−0.01	0.00	0.00	0.00	0.00
M-effect	0.02	0.33	−0.12	−0.18	0.09	0.44	0.45	−0.14

Note: ^a^Years in the range 2008–2010 depending on availability (see Appendix 3)

We now focus on the decompositions of the *StD* changes by age group and by country group (Tables 4 and 5). The age components are aggregated into three broad intervals: childhood ages 0–14; working ages 15–64; and older ages 65+. Table 4 presents the age components of the changes in the *StD* across the whole set of countries, as well as in the between-group *StD* and the within-group *StD*.

**Table 4:** Age components of the total, between-group, and within-group standard deviation change by time periods (in years)

	Males				Females			
	1970–1984	1984–1994	1994–2004	2004–2010^a^	1970–1984	1984–1994	1994–2004	2004–2010^a^
Across all countries								
*All ages:*	*1.96*	*2.09*	*0.36*	*−0.90*	*1.47*	*1.30*	*0.30*	*−0.49*
0–14	0.28	0.02	−0.26	−0.12	0.11	−0.01	−0.17	−0.09
15–64	1.09	1.57	0.18	−0.88	0.59	0.64	0.07	−0.35
65+	0.60	0.50	0.44	0.10	0.78	0.67	0.40	−0.05
Between-group								
*All ages:*	*2.23*	*2.09*	*0.41*	*−0.88*	*1.74*	*1.22*	*0.12*	*−0.48*
0–14	0.35	0.03	−0.26	−0.11	0.24	0.01	−0.19	−0.09
15–64	1.26	1.55	0.25	−0.88	0.65	0.64	0.08	−0.36
65+	0.61	0.51	0.43	0.11	0.85	0.57	0.24	−0.02
Within-group								
*All ages:*	*0.03*	*0.31*	*−0.15*	*−0.19*	*0.09*	*0.44*	*0.45*	*−0.14*
0–14	−0.07	−0.05	−0.02	−0.03	−0.14	−0.04	0.01	−0.02
15–64	−0.05	0.31	−0.22	−0.12	0.07	0.11	−0.02	−0.06
65+	0.15	0.06	0.09	−0.03	0.16	0.37	0.46	−0.07

Note: ^a^Years in the range 2008–2010 depending on availability (see Appendix 3)

**Table 5:** Country-group components of changes in standard deviation by time period (in years)

	Males				Females			
	1970–1984	1984–1994	1994–2004	2004–2010^a^	1970–1984	1984–1994	1994–2004	2004–2010^a^
Across all countries								
*Total:*	*1.96*	*2.09*	*0.36*	*−0.90*	*1.47*	*1.30*	*0.30*	*−0.49*
EME	1.39	0.68	0.92	0.43	1.47	0.78	0.79	0.26
CEE	0.00	−0.02	−0.08	−0.03	−0.15	−0.09	−0.08	−0.02
FSU	0.57	1.43	−0.48	−1.30	0.15	0.61	−0.41	−0.73
Between-group								
*Total:*	*2.23*	*2.09*	*0.41*	*−0.88*	*1.74*	*1.22*	*0.12*	*−0.48*
EME	1.57	0.69	0.97	0.46	1.78	0.70	0.68	0.35
CEE	−0.01	−0.03	−0.09	−0.03	−0.22	−0.11	−0.10	−0.03
FSU	0.67	1.44	−0.46	−1.31	0.17	0.63	−0.46	−0.80
Within-group								
*Total:*	*0.03*	*0.31*	*−0.15*	*−0.19*	*0.09*	*0.44*	*0.45*	*−0.14*
EME	0.06	0.07	−0.07	−0.06	0.07	0.35	0.42	−0.11
CEE	0.03	0.02	0.04	0.01	0.01	0.03	0.03	0.00
FSU	−0.06	0.22	−0.12	−0.14	0.01	0.06	0.01	−0.03

Note: ^a^Years in the range 2008–2010 depending on availability (see Appendix 3)

Decompositions of the *overall* and the *between-group* disparity measures in Table 4 suggest that among males the sharp rise in the degree of divergence between 1970 and 1994 was largely determined by increases in the between-group mortality differences at working ages (15–64) followed by older ages (65+). Among females, the older age group contributed more than the working age group to the between-group mortality differences. Among both males and females, contributions of the mortality changes were much smaller for the childhood age group, but they were also positive (pro-divergence). In 1995–2004, the between-group mortality differences and the overall increases in the *StD* were produced by the older age group and (to a lesser extent) by the working age group. The childhood age group made small and negative (pro-convergence) contributions to the between-group mortality differences and to the overall *StD*.

After 2004, the *overall* and the *between-group StD* values declined. The decompositions show that in this period the *StD* decrease was produced by the working age group and (to a lesser extent) the childhood age group. Among males, the mortality changes at older ages worked against convergence; while among females, the respective components were low.

Compared to the changes in the between-group disparity, the changes in the *within-group disparity* were smaller, especially in 1970–1994, when they were 10 times smaller. The increases in the between-group *StD* coincided with decreases in the within-group *StD* in 1970–1984 among males and females, and in 1984–1994 among males. In 1970–1984, the within-group convergence was produced by the childhood age group (females and males) and the working age group (males only). In 1985–1994, the within-group convergence among males was determined by the working age group. Between 1984 and 2004 the within-group *StD* was nearly unchanged among males, but the within-group disparities increased considerably among females. After 2004, the within-group *StD* declined somewhat due to mortality changes at working and older ages.

Table 5 shows an unusual decomposition of changes in the overall, between-group, and within group disparities by country group. It suggests that the *overall* and the *between-group dispersion* changes (upper part of the table) were largely driven by EME and FSU groups. The part of the CEE group was much smaller due to its lower population weight and smaller temporal changes in life expectancy. Steep life expectancy increase in the EME group leading to widening of the gap between this group and FSU was contributing to the life expectancy divergence throughout the entire period. In the 1970s and especially in the early 1990s, deterioration in FSU largely contributed to the life expectancy divergence. In 2005–2010, life expectancy gains in the FSU produced negative (pro-convergence) contributions to the overall and the between-group *StD* change.

Although changes in the *within-group dispersion* were moderate, there was still a substantial rise in the female within-group disparity from 1985 to 2004, which was largely caused by the growing differences within the EME group.

## Discussion

Although worldwide the average life expectancy has been increasing steadily for many decades, this positive trend has shown heterogeneity across countries. This variation can be investigated using the convergence-divergence framework, which helps to explain interchangeable stages of mortality divergence and convergence [28]. The present study looked solely at developed countries to avoid mixing populations with dramatically different mortality patterns [40].

Our aim was to examine changes in the amount of inter-country lifetime disparity over the last four decades and to assess relative importance of the former East–West geopolitical divide and of temporal changes in the mortality distribution by age and country-group for changes in the amount of disparity. To do this we adapted the general stepwise replacement algorithm for linking changes in countries’ age-specific mortality rates so that it was able to deal with changes in cross-country variance. This method is appropriate in an analysis of temporal changes in an aggregate demographic or public health measure based on a set of populations, rather than on a single population. While the conventional decomposition method permits one to assess the effects of age-specific mortality changes on the total change in life expectancy at birth in a single population, the method used here allowed us to assess the effects of simultaneous changes in numerous country-age-specific mortality rates on the total change in an aggregate scalar measure (the standard deviation of the length of life). This elaborated method is generally applicable to the decomposition of temporal changes in any aggregate index based on a set of populations.

### Limitations

The set of countries under study did not include all developed countries as defined by the UN classification. Although 12 developed countries (Andorra, Bosnia and Herzegovina, Faeroe Islands, Greece, Israel, Macedonia, Lichtenstein, Moldova, Romania, Slovenia, San Marino, Serbia) were missing, there are two reasons why this omission is unlikely to have biased our findings. First, the life expectancy estimates for every country of the world by the UN Population Division signal that the life expectancy values in all of the missing countries are close to the group-specific average life expectancy levels either in the EME or the CEE groups [41]. Second, in 2010 the missing countries had a total population of 62 million, which constitutes only 4.8 % of the total population of all developed countries.

Although we recognize that the usefulness of decomposition analysis is higher if it includes decomposition by causes of death, inclusion of this additional dimension would lead to necessity to carry out first another study to address the cause-of-death inconsistency across time (accounting for changes in ICD revisions) and other changes in diagnostics and coding practices [42, 43]. Therefore, as a first step in using these elaborated methods it was decided to focus on the decomposition for all causes combined.

### Principal findings

Our substantive results show the striking growth in life expectancy variance across the developed countries from the early 1970s to the early 2000s with the variance being much greater for males than for females. Despite the convergence in the period 2005–2010, life expectancy disparities across the developed countries are still enormous with the cross-country *StD* values being three times higher in 2010 than in 1970.

The rising between-group variance constitutes a core part of this phenomenon. Even 25 years after the fall of the Berlin Wall, a large fraction of the variance of the length of life is attributable to the East–West divide. This historical geopolitical gap between the two parts of the developed world grossly determines the life expectancy of the people living in respective countries. The influence of the politics of the 20th century on survival and longevity [12] has thus persisted far longer than was anticipated 25 years ago.

The overall and the between-group lifetime variance are much higher among males than among females. Rise in *between-group disparity* was mainly fueled by widening of the East–West mortality gap at ages 15 to 64 years among males and at ages 15+ years among females. Both the continuous progress in EME and the inconsistent and largely unfavorable changes in FSU contributed to the disparity. The remarkable mortality excess in adult age mortality (especially among males) in FSU and CEE compared to EME is in line with findings of earlier research on Russia and other ex-communist countries [10, 44].

While a large part of the between-group variance was determined by the FSU countries, the within-group variance was dominated by the EME. Unlike the between-group variance, the within-group variance and its changes were greater among females than they were among males starting in the late 1990s. The onset of this pattern coincided with the time when nearly all female mortality was already concentrated at old ages and reduction of old age mortality became an ultimate condition of further longevity progress. It appears that even across the EME countries there are substantial differences with respect to success in reduction of female mortality at old ages. This was examined by Meslé and Vallin, [24] who provided detailed comparison of components and possible drivers of old-age mortality trends in several advanced countries. According to their convergence-divergence theory, the growing disparity in longevity among women should be related to emergence of a new health challenge. This time, the challenge is connected to a hard transition from reduction of cardiovascular death at younger old ages to reduction of death from multiple pathologies at advanced ages.

Among males, the moderate changes in the within-group variance were mostly related to the FSU group and to mortality at working ages.

In the second half of the 2000s, both the between-group and the within-group parts of the inter-country variance declined due to mortality convergence at ages 15 to 64 in the FSU group and (to a much smaller extent) to mortality convergence at ages 65+ in the EME group. It is worth noting that the life expectancy gap between the CEE and the EME countries, which was shrinking over the 1990s as mortality in the CEE countries steeply declined, stabilized in the 2000s due to a slowdown in the positive trends in the CEE group.

### Sensitivity analyses

The population-weighted variance metric used in this study is focused on individuals since it accounts for numbers of humans who are exposed to higher or lower death hazard in different places. There is, however, a disadvantage due to a low sensitivity of the metrics to mortality and mortality changes in countries with small populations. In addition, one may be particularly interested in to what extent results of our analysis depend on the USA, a country with the largest population size, about 300 million, which is somewhat lagging behind within the group of EME countries over the last two decades.

To evaluate how influential the very large contribution of the USA was to our findings we repeated all computations with this country excluded (outcomes not shown here but can be provided upon request). All in all, this change produces a minor impact on the results pertaining to the overall lifetime disparity and its changes. Average life expectancy in the EME group becomes slightly higher and its population weight becomes slightly lower, which results in slightly lower between-group and overall standard deviations. More important impacts are seen in the within-group standard deviation which becomes lower (especially for females) by about one-third in the 1990s and the 2000s. This happens mostly due to a diminished weight of the EME group in the within-group disparity. The temporal increase in the within-group disparity among females becomes somewhat smaller, contribution of ages 65+ diminishes by about 40 %, and contribution of EME to this increase diminishes by about one-fourth.

Use of the population-unweighted data produces a more visible change in the calculation outcomes (see Appendix 6 for detailed tables). This metric expresses the amount of inter-country difference in length of life among countries irrespective of their population sizes, counting each country as one unit.

With unweighted data, life expectancy of EME slightly increases and life expectancy of FSU increases substantially – by 2.5–3.5 years in the 1990s–2000s. The overall *StD* becomes substantially lower and its increase from 1970 to 2004 and decrease from 2004 to 2010 are becoming much less pronounced. All age components of the disparity changes are becoming substantially smaller. Contributions of ages 15 to 64 are still decisive for the overall and the between-group variance changes, especially among males, as well as contributions of ages 65+ to the within-group variance changes among females. Although contributions of the CEE group to the disparity changes increase, they are still substantially lower compared to FSU and EME.

## Conclusions

Over most of the period 1970–2010 there was an overall tendency for life expectancy to diverge across the 36 developed countries examined. This was driven primarily by the growing gap between the EME and FSU groups due to diverse changes in mortality at working ages (especially among males) and at older ages. Within the EME group, divergence occurred in the 1990s and the early 2000s due to uneven progress with respect to female mortality at ages 65+. The within-group lifetime disparity rise among women was substantially dependent on the USA. Old-age and working-age mortality rates are still substantially higher in some places than in others, generating important variations in length of life across developed countries. This signals that many countries have significant scope for further health improvement, even at the existing levels of medical technology and economic wealth.

The inter-country mortality disparities point to the major health challenges, which even many developed countries continue to face. These disparities still persist in large part because four decades ago countries had moderately differing levels of length of life but have subsequently shown very variable capacity to address major health challenges [37]. Further monitoring and analysis of cross-country variation in longevity and survival and identification of the factors associated with success or failure in the fight to extend longevity should be a priority.

## Appendix 1

**Table 6:** Countries and country groups under study

Established market economies	Central and Eastern Europe	Former Soviet Union
Australia	Bulgaria	Belarus
Austria	Czech Republic	Estonia
Belgium	East Germany	Latvia
Canada	Hungary	Lithuania
Denmark	Poland	Russian Federation
England & Wales	Slovakia	Ukraine
Finland		
France		
Iceland		
Ireland		
Italy		
Japan		
Luxembourg		
Netherlands		
New Zealand		
Northern Ireland		
Norway		
Portugal		
Scotland		
Spain		
Sweden		
Switzerland		
United States of America		
West Germany		

## Appendix 2

**Table 7:** Life expectancy at birth in selected years (in years)

	Males					Females				
	1970	1984	1994	2004	2010	1970	1984	1994	2004	2010
*Established market economies including:*	*68.18*	*72.08*	*73.91*	*76.56*	*77.90*	*74.75*	*78.75*	*80.43*	*82.18*	*83.13*
Australia^a^	67.48	72.66	74.98	78.45	79.73	74.29	79.21	80.89	83.29	84.17
Austria	66.49	69.96	73.10	76.41	77.71	73.42	77.14	79.61	82.09	83.13
Belgium	67.82	70.92	73.34	75.96	77.38	74.13	77.76	79.97	81.82	82.63
Canada^a^	69.34	73.01	74.87	77.70	79.02	76.30	79.79	80.89	82.45	83.40
Denmark	70.87	71.63	72.74	75.13	77.11	75.94	77.61	78.06	79.78	81.29
England & Wales	68.89	71.86	74.17	76.89	78.62	75.19	77.72	79.45	81.21	82.53
Finland^a^	66.15	70.47	72.79	75.30	76.51	74.41	78.76	80.13	82.24	83.12
France	68.38	71.13	73.64	76.69	78.06	75.82	79.35	81.88	83.86	84.71
Iceland	70.70	74.75	77.09	78.93	79.73	77.24	80.19	81.31	82.91	83.78
Ireland^a^	68.57	70.88	73.04	76.07	77.26	73.31	76.53	78.63	81.02	82.22
Italy^a^	68.70	72.12	74.47	78.08	79.21	74.55	78.75	81.02	83.77	84.21
Japan	69.32	74.62	76.60	78.61	79.56	74.69	80.30	82.94	85.59	86.34
Luxembourg^a^	66.35	69.54	73.13	75.91	78.05	72.91	76.55	79.41	82.14	82.88
Netherlands^a^	70.82	72.95	74.56	76.87	78.55	76.52	79.67	80.28	81.41	82.63
New Zealand^b^	68.15	71.16	74.15	77.33	78.39	74.49	77.67	79.66	81.42	82.33
Northern Ireland	67.73	70.35	73.17	76.00	77.16	73.67	76.73	78.64	80.71	81.69
Norway^a^	70.98	72.91	74.86	77.49	78.62	77.31	79.49	80.62	82.30	83.03
Portugal	63.90	69.36	72.13	74.92	76.73	70.15	76.44	79.25	81.77	83.04
Scotland	67.13	69.86	72.09	74.25	76.15	73.42	75.89	77.69	79.38	80.61
Spain	69.31	73.20	74.48	76.99	79.01	74.87	79.68	81.71	83.67	85.03
Sweden	72.24	73.82	76.05	78.33	79.50	77.22	79.92	81.34	82.61	83.44
Switzerland	70.02	73.34	75.13	78.46	80.04	76.16	80.02	81.70	83.49	84.37
United States of America	67.02	71.12	72.40	75.02	76.37	74.68	78.18	79.06	80.10	81.20
West Germany	67.32	71.24	73.51	76.54	77.92	73.65	77.89	79.80	81.94	82.72
*Central and Eastern Europe including:*	*67.00*	*67.32*	*67.98*	*71.27*	*72.46*	*73.06*	*74.68*	*76.19*	*79.03*	*79.94*
Bulgaria	69.11	68.34	67.16	69.01	70.28	73.51	74.37	74.73	76.18	77.22
Czech Republic	66.02	67.28	69.45	72.59	74.41	73.00	74.44	76.58	79.18	80.62
East Germany	68.12	69.62	70.69	75.27	76.69	73.31	75.39	78.16	81.73	82.60
Hungary^a^	66.32	64.95	64.96	68.70	70.18	72.10	73.10	74.41	77.14	78.20
Poland^a^	66.44	66.82	67.44	70.61	71.48	73.14	74.92	76.05	79.18	79.90
Slovakia^a^	66.66	66.73	68.25	70.32	71.36	72.94	74.84	76.44	78.03	78.95
*Former Soviet Union including:*	*64.26*	*62.67*	*58.97*	*59.98*	*63.46*	*73.88*	*73.41*	*71.75*	*72.86*	*74.95*
Belarus	67.92	65.42	63.41	63.11	64.57	76.04	75.49	74.36	74.96	76.45
Estonia	65.47	64.57	60.62	66.40	70.57	74.55	74.26	72.95	77.92	80.49
Latvia	65.64	64.05	58.71	65.58	67.42	74.25	74.44	72.21	76.02	77.38
Lithuania	66.84	65.35	62.53	66.28	67.52	75.08	75.53	74.75	77.72	78.73
Russian Federation	63.09	61.68	57.39	58.83	62.90	73.47	72.95	71.06	72.27	74.73
Ukraine^a^	66.52	64.59	62.36	61.96	64.32	74.48	74.08	72.93	73.58	74.79
*All countries*	*67.31*	*69.85*	*70.59*	*73.27*	*75.10*	*74.42*	*77.29*	*78.33*	*80.18*	*81.41*
*Max-min range*	*9.14*	*13.07*	*19.70*	*20.10*	*17.15*	*7.16*	*7.35*	*11.88*	*13.31*	*11.60*
*Standard deviation*	*2.04*	*4.00*	*6.09*	*6.45*	*5.55*	*1.00*	*2.47*	*3.77*	*4.06*	*3.57*

Note: ^a^Data of 2009 used as the last point; ^b^Data of 2008 used as the last point

## Appendix 3

**Table 8:** Population size in selected years (in millions)

	Males					Females				
	1970	1984	1994	2004	2010	1970	1984	1994	2004	2010
*Established market economies including:*	*325.9*	*361.9*	*387.9*	*414.4*	*431.5*	*342.5*	*380.3*	*405.7*	*431.7*	*448.5*
Australia^a^	6.3	7.8	8.9	10.0	10.9	6.3	7.8	9.0	10.1	11.0
Austria	3.5	3.6	3.8	4.0	4.1	3.9	4.0	4.1	4.2	4.3
Belgium	4.7	4.8	4.9	5.1	5.3	4.9	5.1	5.2	5.3	5.6
Canada^a^	10.7	12.7	14.3	15.8	16.7	10.7	12.9	14.6	16.1	17.0
Denmark	2.4	2.5	2.6	2.7	2.8	2.5	2.6	2.6	2.7	2.8
England & Wales	23.8	24.2	24.9	26.0	27.4	25.2	25.5	26.3	27.1	28.3
Finland^a^	2.2	2.4	2.5	2.6	2.6	2.4	2.5	2.6	2.7	2.7
France	24.8	26.8	28.0	29.4	30.5	26.0	28.2	29.6	31.3	32.5
Iceland	0.1	0.1	0.1	0.1	0.2	0.1	0.1	0.1	0.1	0.2
Ireland^a^	1.5	1.8	1.8	2.0	2.2	1.5	1.8	1.8	2.0	2.2
Italy^a^	25.9	27.6	27.6	28.2	29.2	27.4	29.2	29.4	30.0	31.0
Japan	50.5	58.7	60.9	61.6	61.6	52.4	60.7	63.2	64.5	64.8
Luxembourg^a^	0.2	0.2	0.2	0.2	0.2	0.2	0.2	0.2	0.2	0.3
Netherlands^a^	6.5	7.1	7.6	8.1	8.2	6.5	7.3	7.8	8.2	8.4
New Zealand^b^	1.4	1.6	1.8	2.0	2.1	1.4	1.6	1.8	2.1	2.2
Northern Ireland	0.8	0.8	0.8	0.8	0.9	0.8	0.8	0.8	0.9	0.9
Norway^a^	1.9	2.0	2.1	2.3	2.4	1.9	2.1	2.2	2.3	2.4
Portugal	4.1	4.8	4.8	5.1	5.1	4.6	5.2	5.2	5.4	5.5
Scotland	2.5	2.5	2.5	2.4	2.5	2.7	2.7	2.6	2.6	2.7
Spain	16.5	18.8	19.4	21.1	23.0	17.3	19.5	20.2	21.8	23.6
Sweden	4.0	4.1	4.3	4.5	4.7	4.0	4.2	4.4	4.5	4.7
Switzerland	3.0	3.1	3.4	3.6	3.9	3.2	3.3	3.6	3.8	4.0
United States of America	99.3	114.7	128.5	143.7	152.0	104.7	121.2	134.4	148.8	157.1
West Germany	29.1	29.2	32.0	33.1	33.1	32.0	31.9	33.8	34.6	34.4
*Central and Eastern Europe including:*	*39.9*	*42.9*	*42.9*	*41.9*	*41.6*	*42.8*	*45.6*	*45.5*	*44.5*	*44.1*
Bulgaria	4.2	4.4	4.1	3.8	3.6	4.2	4.5	4.3	4.0	3.8
Czech Republic	4.7	5.0	5.0	5.0	5.1	5.1	5.3	5.3	5.3	5.3
East Germany	7.9	7.9	7.5	7.2	7.0	9.2	8.8	8.0	7.5	7.2
Hungary^a^	5.0	5.1	4.9	4.8	4.8	5.3	5.5	5.4	5.3	5.3
Poland^a^	15.8	18.0	18.7	18.5	18.4	16.7	18.9	19.7	19.7	19.7
Slovakia^a^	2.2	2.5	2.6	2.6	2.6	2.3	2.6	2.7	2.8	2.8
*Former Soviet Union including:*	*88.1*	*97.3*	*101.1*	*96.4*	*94.2*	*105.4*	*113.0*	*115.1*	*111.4*	*109.6*
Belarus	4.2	4.6	4.8	4.5	4.4	4.9	5.3	5.4	5.2	5.1
Estonia	0.6	0.7	0.7	0.6	0.6	0.7	0.8	0.8	0.7	0.7
Latvia	1.1	1.2	1.2	1.0	1.0	1.3	1.4	1.4	1.2	1.1
Lithuania	1.5	1.7	1.7	1.6	1.4	1.7	1.9	1.9	1.8	1.7
Russian Federation	59.4	65.8	68.9	66.8	65.6	70.9	76.2	78.0	77.0	76.3
Ukraine^a^	21.4	23.3	23.9	21.8	21.1	25.9	27.5	27.6	25.4	24.7
*All countries*	*453.8*	*502.1*	*531.9*	*552.7*	*567.3*	*490.8*	*538.9*	*566.2*	*587.7*	*602.2*

Note: ^a^Data of 2009 used as the last point; ^b^Data of 2008 used as the last point

## Appendix 4

**Brief summary of the general stepwise replacement algorithm**

The idea of this method is to present decomposition of the total change in an index *f*(.) as a sequence of replacement of its arguments. Let *f*(.) depend on (say) three covariates *a*, *b*, and *c* which are defined at time points 1 and 2. The difference *f*(*a*_2_,*b*_2_,*c*_2_)-*f*(*a*_1_,*b*_1_,*c*_1_) can be presented as a sum of effects of a sequence of replacements:

$$ f\left({a}_2,{b}_2,{c}_2\right)-f\left({a}_1,{b}_1,{c}_1\right)=\varDelta f\left({a}_2\to {a}_1,{b}_1,{c}_1\right)+\varDelta f\left({a}_2,{b}_2\to {b}_1,{c}_2\right)+\varDelta f\left({a}_2,{b}_2,{c}_2\to {c}_1\right) $$

with the *delta* components

$$ \varDelta f\left({a}_2\to {a}_1,{b}_1,{c}_1\right)=f\left({a}_2,{b}_1,{c}_1\right)-f\left({a}_1,{b}_1,{c}_1\right); $$

$$ \varDelta f\left({a}_2,{b}_2\to {b}_1,{c}_1\right)=f\left({a}_2,{b}_2,{c}_1\right)-f\left({a}_2,{b}_1,{c}_1\right); $$

$$ \varDelta f\left({a}_2,{b}_2,{c}_2\to {c}_1\right)=f\left({a}_2,{b}_2,{c}_2\right)-f\left({a}_2,{b}_2,{c}_1\right). $$

Each of these three components is equal to the contribution of the shift of the value of respective covariate from point 1 to point 2. Note that once the value of the independent variable (say *a*) is shifted from *a*_1_ to *a*_2_, it remains equal to *a*_2_ when delta-components of covariates that come later in the replacement sequence are calculated.

The three components given above correspond to the replacement sequence *a-b-c*. It is possible, however, to move from *a*_1_*b*_1_*c*_1_ to *a*_2_*b*_2_*c*_2_ using other pathways. For example, it is possible to carry out the replacement sequence *b-a-c* (*b*_2_ → *b*_1_, *a*_2_ → *a*_1_, *c*_2_ → *c*_1_). The number of all possible replacement sequences equals 6 (all possible permutations among three elements).

For a non-linear function *f*(*a*, *b*, *c*), values of the component produced by a movement from point 1 to point 2 for the same covariate in different replacement sequences are not exactly equal to each other. For example, the *a*-component *Δf*(*a*_2_ → *a*_1_, *b*_2_, *c*_1_) = *f*(*a*_2_, *b*_2_, *c*_1_) − *f*(*a*_1_, *b*_2_, *c*_1_) in sequence *b-a-c* is not exactly the same compared to the *a*-component same component in sequence *a-b-c Δf*(*a*_2_ → *a*_1_, *b*_1_, *c*_1_) = *f*(*a*_2_, *b*_1_, *c*_1_) − *f*(*a*_1_, *b*_1_, *c*_1_).

Thus, the stepwise replacement has to be carried out for all possible replacement sequences (permutations) and the final components are to be computed as averages of the components’ values over all these sequences [29, 45, 46]. In our example it means that every component effect (*a*, *b*, or *c*) should be calculated as average of six respective sequence-specific component values.

The stepwise replacement algorithm with the full run across all replacement sequences can be applied for decompositions concerning countries or regions as we have done here, or many important socio-demographic variables with small numbers of categories such as sex, cause of death by broad diagnostic groups, education or socioeconomic status by aggregate categories, birth order from 1 to 5+ and other.

However, there is an obvious difficulty when it comes to the age variable, which has high number of categories: about 20 and 100 in abridged and complete life tables, respectively, and about 30 in a fertility tables. Therefore, it was suggested to compute the age components by using the replacement sequence that is running in ascending order of ages [29]. Such an approach guarantees that the stepwise replacement algorithm’s results are exactly equal to results of the most used analytical decomposition formulae by Andreev (1982), Arriaga (1984), and Pressat (1985) in the case of life expectancy.

## Appendix 5

**Stepwise replacement algorithm for decomposition by age and country group**

Similar to decomposition by age, mortality and population composition (M- and P-effects), one can estimate the contribution of a country (country group) to the total change. Following the logic of M-P decomposition, we estimate country-specific contributions within age components. In other words, every age-specific contribution has to be split by country effects.

Below we provide a general formal description for the case of three populations which can be extended to any number of countries. It is important to note here that the calculation of country effects is a resource-consuming task which requires in case of *K* countries 2*K*! calculations of the index function *F*(.) for every age group. Thus, even for the case of *K* = 10 this task might be not affordable.

Let the index function *F*(.) depends on matrices **M** and **P**. Following the general rule of the age decomposition the replacement in matrices **M** and **P** progresses from the youngest age 0 to the oldest age 95+. Let **M**_*k*_^[*x*]^(*t*_0_, *T*) and **P**_*k*_^[*x*]^(*t*_0_, *T*) be matrices containing elements *m*_*y*,*i*_(*T*) and *p*_*y*,*i*_(*T*) at ages 0 ≤ *y* < *x* − *a* and at age *x* - *a* for counties *k*, elements *m*_*y*,*i*_(*t*_0_) and *p*_*y*,*i*_(*t*_0_) at ages *y* ≥ *x* and at age *x* - *a* for counties other than *k*. For example, for *k* = 1,2:

14 $$ {\mathbf{M}}_{1,2}^{\left[\boldsymbol{x}\right]}\left({t}_0,T\right)=\left[\begin{array}{ccc}\hfill {}_1{m}_{0,1}(T)\hfill & \hfill {}_1{m}_{0,2}(T)\hfill & \hfill {}_1{m}_{0,3}(T)\hfill \\ {}\hfill {}_4{m}_{1,1}(T)\hfill & \hfill {}_4{m}_{1,2}(T)\hfill & \hfill {}_4{m}_{1,3}(T)\hfill \\ {}\hfill \cdots \hfill & \hfill \cdots \hfill & \hfill \cdots \hfill \\ {}\hfill {}_5{m}_{x-5,1}(T)\hfill & \hfill {}_5{m}_{x-5,2}(T)\hfill & \hfill {}_5{m}_{x-5,3}\left({t}_0\right)\hfill \\ {}\hfill {}_5{m}_{x,1}\left({t}_0\right)\hfill & \hfill {}_5{m}_{x,2}\left({t}_0\right)\hfill & \hfill {}_5{m}_{x,3}\left({t}_0\right)\hfill \\ {}\hfill \cdots \hfill & \hfill \cdots \hfill & \hfill \cdots \hfill \\ {}\hfill {}_{\infty }{m}_{95,1}\left({t}_0\right)\hfill & \hfill {}_{\infty }{m}_{95,2}\left({t}_0\right)\hfill & \hfill {}_{\infty }{m}_{95,3}\left({t}_0\right)\hfill \end{array}\right], $$

$$ {\mathbf{M}}_0^{\left[x\right]}\left({t}_0,T\right)={\mathbf{M}}_{1,2,3}^{\left[x-a\right]}\left({t}_0,T\right)\;\mathrm{and}\ {\mathbf{M}}_0^{\left[0\right]}\left({t}_0,T\right)=\mathbf{M}\left({t}_0\right), $$

15 $$ {\mathbf{P}}_{1,2}^{\left[\boldsymbol{x}\right]}\left({t}_0,T\right)=\left[\begin{array}{ccc}\hfill \begin{array}{l}{}_1{p}_{0,1}(T)\\ {}{}_4{p}_{1,1}(T)\end{array}\hfill & \hfill \begin{array}{l}{}_1{p}_{0,2}(T)\\ {}{}_4{p}_{1,2}(T)\end{array}\hfill & \hfill \begin{array}{l}{}_1{p}_{0,3}(T)\\ {}{}_4{p}_{1,3}(T)\end{array}\hfill \\ {}\hfill \cdots \hfill & \hfill \cdots \hfill & \hfill \cdots \hfill \\ {}\hfill {}_5{p}_{x-5,1}(T)\hfill & \hfill {}_5{p}_{x-5,2}(T)\hfill & \hfill {}_5{p}_{x-5,3}\left({t}_0\right)\hfill \\ {}\hfill {}_5{p}_{x,1}\left({t}_0\right)\hfill & \hfill {}_5{p}_{x,2}\left({t}_0\right)\hfill & \hfill {}_5{p}_{x,3}\left({t}_0\right)\hfill \\ {}\hfill \cdots \hfill & \hfill \cdots \hfill & \hfill \cdots \hfill \\ {}\hfill {}_{\infty }{p}_{95,2}\left({t}_0\right)\hfill & \hfill {}_{\infty }{p}_{95,2}\left({t}_0\right)\hfill & \hfill {}_{\infty }{p}_{95,3}\left({t}_0\right)\hfill \end{array}\right], $$

$$ {\mathbf{P}}_0^{\left[x\right]}\left({t}_0,T\right)={\mathbf{P}}_{1,2,3}^{\left[x-a\right]}\left({t}_0,T\right)\;\mathrm{and}\kern0.37em {\mathbf{P}}_0^{\left[0\right]}\left({t}_0,T\right)=\mathbf{P}\left({t}_0\right), $$

One step in the replacement sequence includes replacement pertaining a single age group [*x*,*x* + *a*). The elements corresponding to this age group in the two matrices **M** and **P** should be replaced simultaneously for every country *k*. The effect of this replacement on the value of *F* should be computed as an average effect of the replacement a country-specific element by all possible permutations. Using the notation given in (14) and (15), the effect of country 1 within the age component *x* is:

16 $$ \begin{array}{l}{\varDelta}_1^x\left({t}_0,T\right)=\frac{1}{4}\left\{\left[F\left({\mathbf{M}}_1^{\left[x+a\right]},{\mathbf{P}}_1^{\left[x+a\right]}\right)-F\left({\mathbf{M}}_0^{\left[x+a\right]},{\mathbf{P}}_0^{\left[x+a\right]}\right)\right]\right.\\ {}\kern3.48em +\left[F\left({\mathbf{M}}_{1,2}^{\left[x+a\right]},{\mathbf{P}}_{1,2}^{\left[x+a\right]}\right)-F\left({\mathbf{M}}_2^{\left[x+a\right]},{\mathbf{P}}_2^{\left[x+a\right]}\right)\right]\\ {}\kern3.48em +\left[F\left({\mathbf{M}}_{1,3}^{\left[x+a\right]},{\mathbf{P}}_{1,3}^{\left[x+a\right]}\right)-F\left({\mathbf{M}}_3^{\left[x+a\right]},{\mathbf{P}}_3^{\left[x+a\right]}\right)\right]\\ {}\kern3.36em \left.\left.+\left[F\left({\mathbf{M}}_{1,2,3,}^{\left[x+a\right]},{\mathbf{P}}_{1,2,3,}^{\left[x+a\right]}\right)-F\left({\mathbf{M}}_{2,3,}^{\left[x+n\right]},{\mathbf{P}}_{2,3,}^{\left[x+a\right]}\right)\right]\right\}\right|\end{array} $$

The effects of second and third countries are:

17 $$ \begin{array}{l}{\Delta}_2^x\left({t}_0,T\right)=\frac{1}{4\ }\left\{\left[F\left({\mathbf{M}}_2^{\left[x+a\right]},{\mathbf{P}}_2^{\left[x+a\right]}\right)-F\left({\mathbf{M}}_0^{\left[x+a\right]},{\mathbf{P}}_0^{\left[x+a\right]}\right)\right]\right.\\ {}\kern3.72em +\left[F\left({\mathbf{M}}_{1,2}^{\left[x+a\right]},{\mathbf{P}}_{1,2}^{\left[x+a\right]}\right)-F\left({\mathbf{M}}_1^{\left[x+a\right]},{\mathbf{P}}_1^{\left[x+a\right]}\right)\right]\\ {}\kern3.72em +\left[F\left({\mathbf{M}}_{2,3}^{\left[x+a\right]},{\mathbf{P}}_{2,3}^{\left[x+a\right]}\right)-F\left({\mathbf{M}}_3^{\left[x+a\right]},{\mathbf{P}}_3^{\left[x+a\right]}\right)\right]\;\\ {}\kern3.72em \left.+\left[F\left({\mathbf{M}}_{1,2,3}^{\left[x+a\right]},{\mathbf{P}}_{1,2,3}^{\left[x+a\right]}\right)-F\left({\mathbf{M}}_{2,3}^{\left[x+a\right]},{\mathbf{P}}_{2,3}^{\left[x+a\right]}\right)\right]\right\}\end{array} $$

18 $$ \begin{array}{l}{\Delta}_3^x\left({t}_0,T\right)=\frac{1}{4\ }\left\{\left[F\left({\mathbf{M}}_3^{\left[x+a\right]},{\mathbf{P}}_3^{\left[x+a\right]}\right)-F\left({\mathbf{M}}_0^{\left[x+a\right]},{\mathbf{P}}_0^{\left[x+a\right]}\right)\right]\right.\\ {}\kern3.6em +\left[F\left({\mathbf{M}}_{2,3}^{\left[x+a\right]},{\mathbf{P}}_{2,3}^{\left[x+a\right]}\right)-F\left({\mathbf{M}}_2^{\left[x+a\right]},{\mathbf{P}}_2^{\left[x+a\right]}\right)\right]\\ {}\kern3.6em +\left[F\left({\mathbf{M}}_{1,3}^{\left[x+a\right]},{\mathbf{P}}_{1,3}^{\left[x+a\right]}\right)-F\left({\mathbf{M}}_1^{\left[x+a\right]},{\mathbf{P}}_1^{\left[x+a\right]}\right)\right]\\ {}\kern3.6em \left.+\left[F\left({\mathbf{M}}_{1,2,3}^{\left[x+a\right]},{\mathbf{P}}_{1,2,3}^{\left[x+a\right]}\right)-F\left({\mathbf{M}}_{1,2}^{\left[x+a\right]},{\mathbf{P}}_{1,2}^{\left[x+a\right]}\right)\right]\right\}\end{array} $$

The component corresponding to change in the elementary age group [*x*, *x* + *a*) is equal to the sum of the respective country contributions:

$$ {\Delta}^x={\displaystyle \sum_{k=1}^3}{\Delta}_k^x. $$

The total change in the function *F* between times *t*_0_ and *T* is:

$$ F(T)-F\left({t}_0\right)={\displaystyle \sum_x}{\Delta}^x. $$

## Appendix 6

**Alternative tables of results with the population-unweighted measures**

Tables 9–13 below correspond to Tables 1–5 in the main text.

**Table 9:** Life expectancy at birth and measures of variance for the entire set of countries and for country groups in selected years (in years)

	Males					Females				
	1970	1984	1994	2004	2010^a^	1970	1984	1994	2004	2010^a^
*Overall life expectancy, including:*	*67.83*	*69.78*	*70.82*	*73.64*	*75.22*	*74.45*	*77.09*	*78.29*	*80.41*	*81.52*
EME	68.49	71.78	74.02	76.76	78.18	74.76	78.39	80.16	82.12	83.10
CEE	67.11	67.29	67.99	71.09	72.40	73.00	74.51	76.06	78.57	79.58
FSU	65.91	64.27	60.84	63.69	66.21	74.64	74.46	73.04	75.42	77.10
*Max-Min range between country groups*	2.57	7.51	13.18	13.07	11.97	1.76	3.93	7.12	6.71	6.01
*Overall standard deviation, including:*	*1.97*	*3.30*	*5.22*	*5.22*	*4.86*	*1.51*	*2.19*	*3.06*	*3.03*	*2.80*
EME	*1.87*	*1.52*	*1.32*	*1.29*	*1.14*	*1.61*	*1.35*	*1.28*	*1.40*	*1.27*
CEE	1.12	1.44	1.81	2.26	2.37	0.45	0.72	1.25	1.77	1.74
FSU	1.50	1.25	2.17	2.73	2.56	0.79	0.88	1.24	2.06	2.06

Note: ^a^As in Table 1

**Table 10:** Between- and within-group variance and its distribution by country groups in selected years

	Males					Females				
	1970	1984	1994	2004	2010^a^	1970	1984	1994	2004	2010^a^
*Total cross-country variance, years squared*	*3.89*	*10.91*	*27.27*	*27.28*	*23.59*	*2.29*	*4.81*	*9.36*	*9.20*	*7.86*
*Between-group variance, years squared*	0.99	8.76	24.78	24.08	20.69	0.42	3.38	7.76	6.68	5.56
EME, %	29.3	30.5	27.6	27.0	28.2	15.6	33.3	30.1	29.2	30.2
CEE, %	8.7	11.8	5.4	4.5	6.4	83.0	32.6	10.7	8.5	11.2
FSU, %	62.0	57.7	67.0	68.5	65.4	1.5	34.0	59.2	62.4	58.6
*Between-group standard deviation, years*	*0,99*	*2,96*	*4,98*	*4,91*	*4,55*	*0,65*	*1,84*	*2,79*	*2,58*	*2,36*
*Within-group variance, years squared*	*2.90*	*2.15*	*2.49*	*3.20*	*2.90*	*1.87*	*1.43*	*1.61*	*2.53*	*2.30*
EME, %	80.0	71.6	46.6	34.7	29.9	92.7	84.9	67.8	51.4	47.1
CEE, %	7.2	16.2	21.9	26.6	32.4	1.8	6.0	16.1	20.6	22.0
FSU, %	12.9	12.2	31.5	38.7	37.8	5.5	9.1	16.1	28.0	30.9
*Within-group standard deviation, years*	*1,70*	*1,46*	*1,58*	*1,79*	*1,70*	*1,37*	*1,20*	*1,27*	*1,59*	*1,52*

Note: ^a^As in Table 1

**Table 11:** Contributions of population composition (P-effects) and mortality (M-effects) to changes in standard deviation by time periods (in years)

	Males				Females			
	1970–1984	1984–1994	1994–2004	2004–2010^a^	1970–1984	1984–1994	1994–2004	2004–2010^a^
*Total change in overall StD*	*1.33*	*1.92*	*0.00*	*−0.37*	*0.68*	*0.87*	*−0.03*	*−0.23*
P-effect	0.00	0.00	0.00	0.00	0.00	0.00	0.00	0.00
M-effect	1.33	1.92	0.00	−0.37	0.68	0.87	−0.03	−0.23
*Total change in between-group StD*	*1.97*	*2.02*	*−0.07*	*−0.36*	*1.19*	*0.95*	*−0.20*	*−0.23*
P-effect	0.00	0.00	0.00	0.00	0.00	0.00	0.00	0.00
M-effect	1.97	2.02	−0.07	−0.36	1.19	0.95	−0.20	−0.23
*Total change in within-group StD*	*−0.24*	*0.11*	*0.21*	*−0.08*	*−0.17*	*0.07*	*0.32*	*−0.07*
P-effect	0.00	0.00	0.00	0.00	0.00	0.00	0.00	0.00
M-effect	−0.24	0.11	0.21	−0.08	−0.17	0.07	0.32	−0.07

Note: ^a^As in Table 1

**Table 12:** Age components of the total, between-group, and within-group standard deviation change by time periods (in years)

	Males				Females			
	1970–1984	1984–1994	1994–2004	2004–2010^a^	1970–1984	1984–1994	1994–2004	2004–2010^a^
Across all countries								
*All ages:*	1.33	1.92	0.00	−0.37	0.87	−0.03	−0.23	1.33
0–14	0.04	0.03	−0.15	−0.08	0.01	−0.12	−0.06	0.04
15–64	0.93	1.45	−0.27	−0.47	0.43	−0.09	−0.16	0.93
65+	0.35	0.44	0.42	0.18	0.42	0.19	−0.01	0.35
Between-group								
*All ages:*	1.97	2.02	−0.07	−0.36	0.95	−0.20	−0.23	1.97
0–14	0.26	0.06	−0.16	−0.07	0.05	−0.14	−0.07	0.26
15–64	1.18	1.49	−0.32	−0.47	0.49	−0.15	−0.17	1.18
65+	0.52	0.47	0.41	0.19	0.40	0.09	0.01	0.52
Within-group								
*All ages:*	−0.24	0.11	0.21	−0.08	0.07	0.32	−0.07	−0.24
0–14	−0.23	−0.08	−0.01	−0.03	−0.06	0.00	−0.01	−0.23
15–64	0.03	0.19	0.11	−0.07	0.00	0.09	−0.04	0.03
65+	−0.04	0.01	0.11	0.02	0.13	0.23	−0.03	−0.04

Note: ^a^As in Table 1

**Table 13:** Country-group components of changes in standard deviation by time period (in years)

	Males				Females			
	1970–1984	1984–1994	1994–2004	2004–2010^a^	1970–1984	1984–1994	1994–2004	2004–2010^a^
Across all countries								
*Total:*	1.33	1.92	0.00	−0.37	0.87	−0.03	−0.23	1.33
EME	0.93	0.87	1.10	0.55	0.69	0.80	0.33	0.93
CEE	0.02	−0.05	−0.24	−0.11	−0.21	−0.24	−0.11	0.02
FSU	0.38	1.11	−0.86	−0.80	0.38	−0.59	−0.45	0.38
Between-group								
*Total:*	1.97	2.02	−0.07	−0.36	0.95	−0.20	−0.23	1.97
EME	1.46	0.98	1.17	0.61	0.82	0.87	0.44	1.46
CEE	−0.01	−0.08	−0.28	−0.13	−0.28	−0.32	−0.13	−0.01
FSU	0.51	1.12	−0.96	−0.84	0.40	−0.75	−0.53	0.51
Within-group								
*Total:*	−0.24	0.11	0.21	−0.08	0.07	0.32	−0.07	−0.24
EME	−0.25	−0.12	−0.02	−0.07	−0.05	0.07	−0.07	−0.25
CEE	0.04	0.07	0.09	0.03	0.07	0.09	0.00	0.04
FSU	−0.04	0.17	0.13	−0.04	0.05	0.16	0.00	−0.04

Note: ^a^As in Table 1

## Footnotes

1 We selected all HMD countries classified as developed by the UN for which data were available at least since 1970. Data series for Slovenia and Israel were excluded since they begin after 1970. For thirteen populations which had shorter data series, 2009 (twelve populations) or 2008 (one population) was used as the last data point. For more details, see Appendices 2 and 3.
